# *Dioctophyme renale* in wandering dogs in Pelotas, South of Brasil

**DOI:** 10.1590/S1984-29612022008

**Published:** 2022-02-21

**Authors:** Carolina Buss Brunner, Haide Valeska Scheid, Fabiano da Rosa Venancio, Jéssica Line Farias de Lima, Leonardo Schuler Faccini, Eliza Simone Viégas Sallis, Margarida Buss Raffi

**Affiliations:** 1 Setor de Patologia Veterinária, Departamento de Patologia Clínica Veterinária, Faculdade de Veterinária, Universidade Federal do Rio Grande do Sul – UFRGS, Porto Alegre, RS, Brasil; 2 Laboratório Regional de Diagnóstico, Departamento de Patologia Animal, Faculdade de Veterinária, Universidade Federal de Pelotas – UFPel, Pelotas, RS, Brasil

**Keywords:** Dioctophymosis, stray dog, parasitoses, Dioctofimose, caninos errantes, parasitose

## Abstract

This study aimed to verify the occurrence of *Dioctophyme renale* in stray dogs in the city of Pelotas, in the state of Rio Grande do Sul, Brazil. The Laboratório Regional de Diagnóstico of the Universidade Federal de Pelotas received 146 wandering dogs for necropsy, sent by the City Hall of Pelotas from March 2012 to January 2020. Among the necropsied animals, seventeen dogs (11.64%) were diagnosed with dioctophymosis. Among these dogs, 11 were parasitized with one specimen in the right kidney, two dogs presented two specimens in the right kidney, and in other two dogs, the parasites were in the abdominal cavity. In one dog, two parasites were found in the left kidney; in another dog, both kidneys were parasitized, with two parasites in the right kidney and one in the left kidney. The data obtained in this study showed that the occurrence of *D. renale* in stray dogs in the city of Pelotas is high, and *D. renale* mainly parasitizes the right kidney.

## Resumo

Este trabalho possui como objetivo verificar a ocorrência de *D. renale* em cães errantes do município de Pelotas, no Rio Grande do Sul, Brasil. O Laboratório Regional de Diagnóstico da Universidade Federal de Pelotas recebeu 146 cães errantes para necropsia, encaminhados pela Prefeitura Municipal de Pelotas, no período de março de 2012 a janeiro de 2020. Dentre os cães necropsiados, 17 animais foram diagnosticados com dioctofimose, determinando uma ocorrência de 11,64%. Desses animais, onze estavam parasitados com um exemplar no rim direito, dois animais possuíam dois exemplares no rim direito. Em dois animais, os parasitas estavam na cavidade abdominal; em um animal havia, dois parasitas no rim esquerdo e noutro animal os dois rins estavam parasitados, tendo dois exemplares no rim direito e um exemplar no rim esquerdo. Os dados obtidos neste trabalho permitem concluir que é elevada a ocorrência de *D. renale* nos cães errantes do município de Pelotas, parasitando principalmente o rim direito.

## Introduction

*Dioctophyme renale* (Goeze, 1782) belongs to phylum Nematoda and superfamily Dioctophymoidea; it can reach 100 cm in length and 1.2 cm in diameter (Anderson, 2000). Distributed worldwide and frequently described parasitizing domestic and wild carnivores, *D. renale* was first reported in Brazil in 1860 (Molin) parasitizing a maned wolf (*Chrysocyon brachiurus*) (Leite et al., 2005). However, the most affected animals are dogs, and few are the reports in cattle, horses, pigs, cats, seals, and humans; the latter are considered accidental hosts (Kommers et al., 1999; Leite et al., 2005; Zabott et al., 2012).

The dog is, along with other carnivores, a definitive host but it is also considered terminal because the parasite's life cycle is interrupted, as dogs usually only host one specimen of the parasite (Kommers et al., 1999). The epidemiology of *D. renale* involves an aquatic evolutionary cycle, in which eggs, containing first-stage larvae, must be ingested by oligochaete annelids (*Lumbriculus variegatus*) intermediate hosts. The annelids serve as food for fish, freshwater frogs, and other animals; these animals are classified as paratenic hosts and are part of the food chain of domestic and wild carnivores. The definitive hosts are infected by ingesting the infected annelids, fish, or frogs (Anderson, 2000; Mascarenhas & Müller, 2015).

This study aimed to verify the occurrence of *D. renale* in stray canines in the municipality of Pelotas, in the state of Rio Grande do Sul, Brazil and to report its zoonotic character.

## Materials and Methods

One hundred and forty-six stray dogs from Pelotas had their necropsies performed at the Laboratório Regional de Diagnóstico (LRD), Universidade Federal de Pelotas (UFPel) from March 2012 to January 2020. These animals came from a partnership between the municipality of Pelotas and the Hospital de Clínicas Veterinárias of UFPel (HCV-UFPel) and the LRD/UFPel. The municipality of Pelotas collects aggressive dogs that have bitten someone, or those who are very sick or have died on the street. These animals are referred to the municipal kennel, and, if necessary, sent to the HCV for consultation and treatment. In the case of death, the corpses are sent to the laboratory to carry out the necropsy, with an accompanying brief clinical history when available. During the necropsies, fragments of organs in the abdominal and thoracic cavities and in the brain were collected and fixed in 10% buffered formalin (v/v). After 48 h, the fragments were sectioned, embedded in paraffin, cut into 3-μm-thick sections, and stained using the hematoxylin and eosin (H & E) staining routine.

## Results

**Figure 1:**
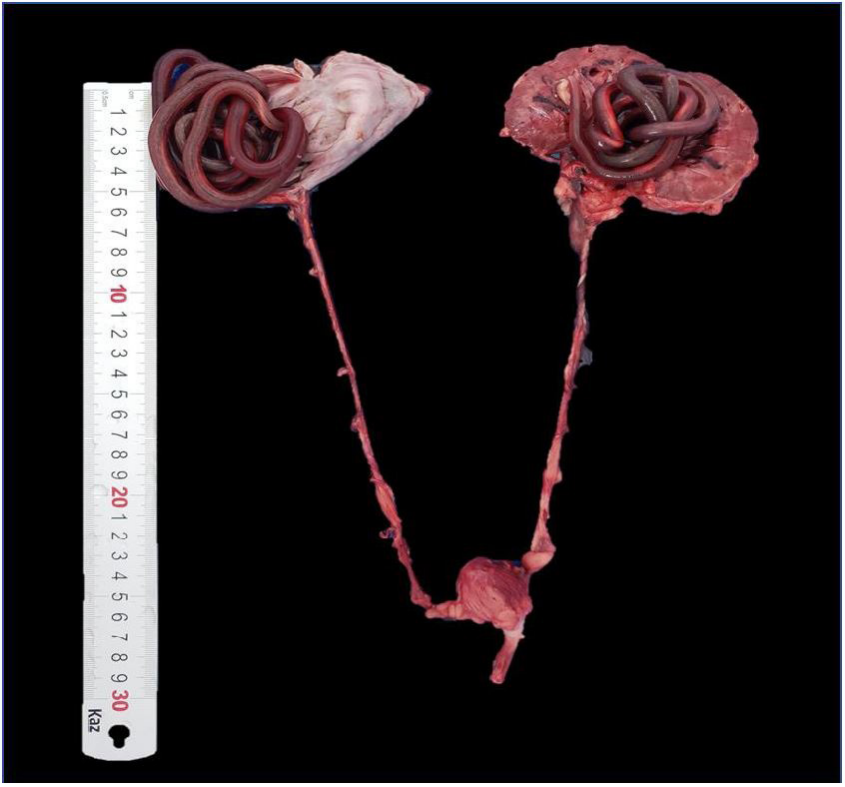
Canine genitourinary tract bilaterally parasitized by *Dioctophyme renale.* In the right kidney there are two pieces of the parasite curled up, dark red and in the left kidney a dark red sample.

**Figure 2:**
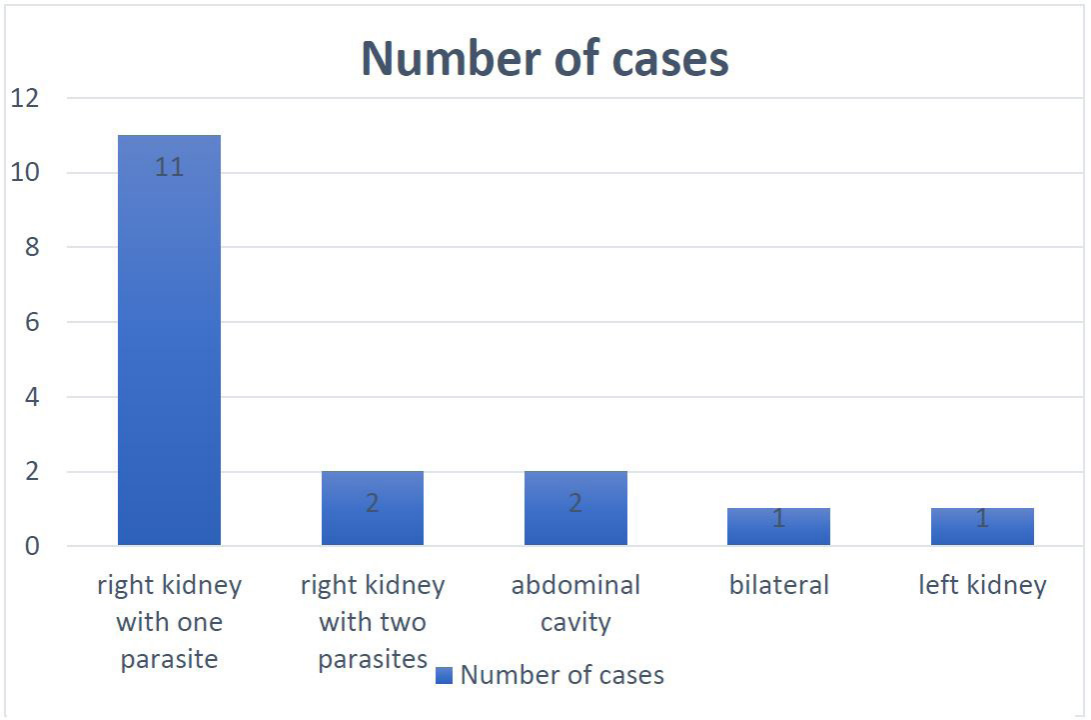
Number of stray dogs parasitized and the affected organ/area by *Dioctophyme renale*.

*Dioctophyme renale* was found in 11.64% (17/146) of the necropsied animals. From the parasitized dogs, 13 (13/17; 76.13%) presented *D. renale* in the right kidney; 11 (11/13) and two (2/13) presented one and two specimens, respectively. One dog (1/17) presented the parasite in the left kidney. In one case (1/17), the parasite was encysted, located next to the lower portion of the urethra; in another case (1/17), the parasite was free in the abdominal cavity. In the last parasitized dog, two specimens were free in the abdominal cavity, two in the right kidney, and one in the left kidney (Figures 1 and 2).

Out of 17 parasitized animals, 88.23% (15/17) were incidental necropsy findings. In only 11.76% (2/17) of the parasitized animals, clinical signs were observed; In one case, the clinical signs were associated with renal failure due to the involvement of the contralateral kidney; whereas in the other case, the signs were associated with severe peritonitis caused by erratic parasitic migration. There was compensatory hypertrophy in the non-parasitized kidney in all animals (16/16).

**Figure 3:**
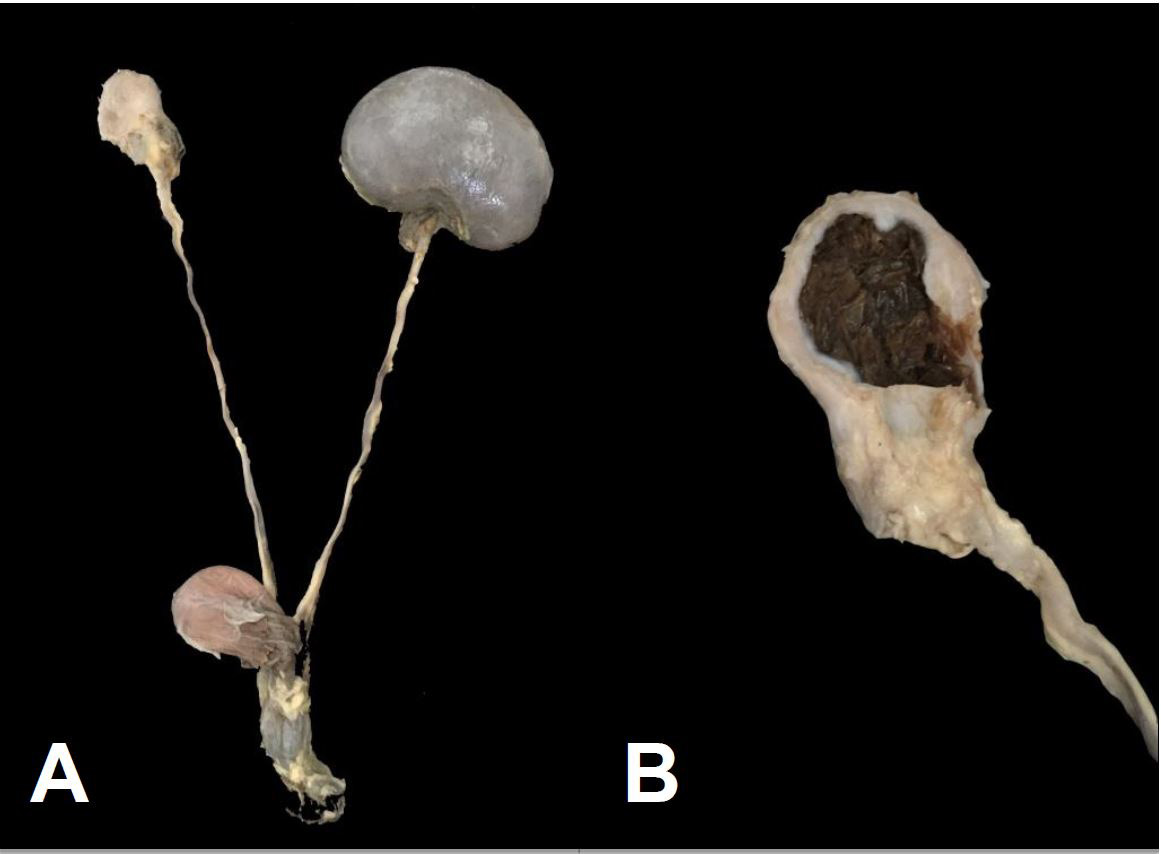
Canine genitourinary tract parasitized by *Dioctophyme renale*. Asymmetric kidneys; right kidney reduced to a fibrous capsule (A). Right kidney: fibrous capsule involving the dissected parasite (B).

The parasitized kidneys showed noticeable destruction of the parenchyma, reducing the organ to a fibrous capsule surrounding the parasite. The kidneys of three animals no longer held the anatomical shape or the normal size of a kidney (Figure 3A), and in one case, only the scar tissue and the dissected parasite were present (Figure 3B). The morphological characteristics of the parasite and the lesions it caused supported the diagnosis of dictiofimosis in the 17 dogs.

**Figure 4:**
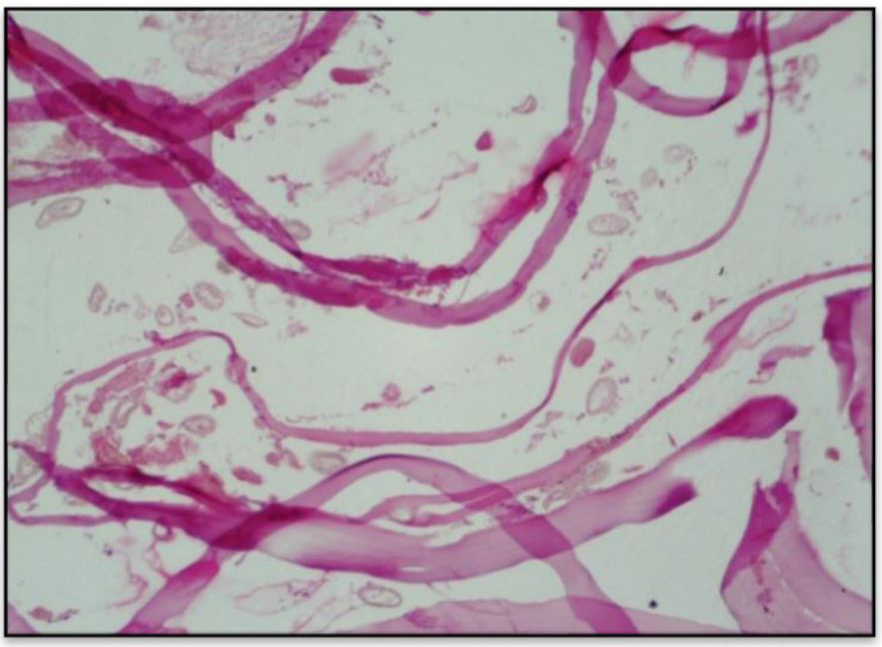
Histological photomicrography of the right kidney with the dissected parasite and eggs (Hematoxylin & Eosin; 100 ×).

Histologically, the parasitized kidneys showed loss of parenchyma due to atrophy, mononuclear inflammatory reaction, glomerulosclerosis, and severe fibrosis of the renal capsule. In some cases, only the capsule was observed as a pouch filled with necro-hemorrhagic fluid containing the parasite. In the kidney in which the dissected parasite was observed, the renal parenchyma was totally lost; only the capsule with fibrotic tissue remained, containing parasite eggs (Figure 4).

## Discussion

In the present study, most dogs parasitized by *D. renale* presented the parasite in one kidney; in most cases, it was the right kidney. There are reports of *D. renale* occurrence in the subcutaneous tissue, scrotal sac, mammary glands, or free in the abdominal cavity, and its preferred location in dogs is still not a consensus (Barriga, 1982; Kommers et al., 1999; Colpo et al., 2007). The location of adult parasites is associated with the location of the larval penetration into the host's digestive tract. Larvae that penetrate the greater curvature of the stomach tend to lodge in the left kidney; larvae that migrate from the duodenum parasites the right kidney, whereas larvae that migrate from the smaller curvature of the stomach can move to the hepatic lobes and become free in the abdominal cavity (Ishizaki et al., 2010; Zabott et al., 2012).

The infected dogs presented compensatory hypertrophy of the kidney contralateral to the parasite; in 88.23% of cases, no clinical signs suggestive of renal impairment was observed. Parasitized animals might have had total atrophy of the affected kidney and remain asymptomatic, and the presence of the parasite becomes an incidental finding during necropsy. However, part of the loss of the renal parenchyma can also be associated with hydronephrosis, a condition caused by obstruction of the internal urethral orifice by adult *D. renale* present in the parasitized kidney (Kommers et al., 1999; Leite et al., 2005). In the present study, 15 (88,23%) dogs presented the parasite as an incidental finding. Only two animals showed clinical signs, one canine with clinical signs of chronic renal failure, and the other one with peritonitis associated with erratic migration of the parasite. In both cases, there was an ultrasound diagnosis suggestive of dioctophymosis.

**Figure 5:**
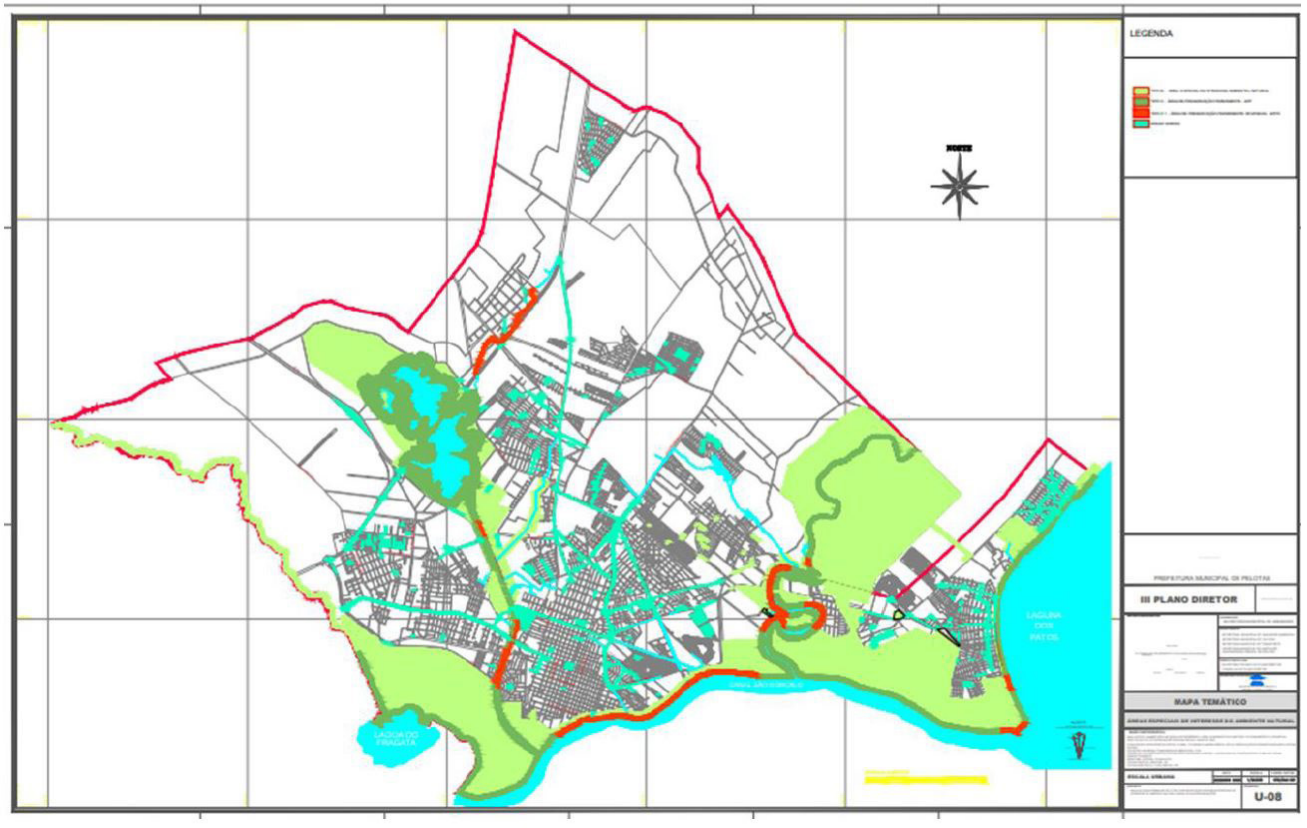
Illustration of the geographical basin of the town of Pelotas.

In this study, occurrence of 11.64% of *D. renale* was observed in wandering dogs in Pelotas, which is higher that the prevalence rates of 0.47%, 0.56, and 1.14% that were reported in the western border and central region of Rio Grande do Sul, and in the state of Parana (Colpo et al., 2007; Kommers et al., 1999; Leite et al., 2005). One of the causes of the high prevalence observed in this study could be the geographical location of the city of Pelotas. Its territorial limits are margins of the Laguna dos Patos, São Gonçalo Stream, Santa Bárbara Dam, Fragata Lagoon, and streams that interconnect these areas (Figure 5) (Pelotas, 2008), leading to favorable conditions for the maintenance and dissemination of the nematode (Mascarenhas & Müller, 2015). The fact that these dogs lived in a town with abundant hydrographic capacity makes them more susceptible to accidental infection through ingestion of paratenic hosts (Pedrassani et al., 2017). In addition, this study focused on stray dogs, which may explain the discrepancies observed by other authors. Stray dogs are unable to obtain adequate water and food, as they have less selective eating habits than housed dogs (Kommers et al., 1999).

The overpopulation of stray dogs has a direct consequence on the high incidence of zoonoses in large population centers (Santana & Oliveira, 2006). Therefore, parasitized dogs can contribute to environmental contamination by excreting urine containing *D. renale* eggs, when they have male and female specimens in the kidney (Perera et al., 2021). Since stray dogs don’t have vaccine control, and diseases are not diagnosed nor treated, they become potential transmitters of pathologies to humans or environmental contaminants (Mascarenhas & Müller, 2015; Pedrassani et al. 2017). The high incidence of dioctophymosis in stray dogs suggests that this disease must be constantly monitored, especially in Pelotas. It is a territory surrounded by large bodies of water, indispensable for the parasite maintenance in the environment, increasing the probability of infection of other animals and humans (Monteiro et al., 2003; Milanelo et al., 2009; Perera et al., 2021). In this context, necropsy data are essential in the epidemiological study of dioctophymosis, especially because of the silent characteristics of the disease.

The population, especially riversiders, should be aware about dioctophymosis, its clinical signs and transmission. They should be informed that this zoonosis is acquired by eating undercooked fish and frog or untreated water contaminated with aquatic annelids and the parasite larvae (Monteiro et al., 2003; Yang et al., 2019). A study reported the presence of this helminth in the skin and kidneys of humans, causing renal colic and hematuria (Chauhan et al., 2016; Norouzi et al., 2017; Yang et al., 2019). There is no treatment for dioctophymosis in humans or animals, and surgical removal of the parasite or parasitic kidney is indicated (Milanelo et al., 2009).

## Conclusion

The data obtained in this study show that the occurrence of *D. renale* in stray dogs in the town of Pelotas is high, and they mainly parasitize the right kidney.
